# Biceps Femoris Injury a Rarity: A Case Report

**DOI:** 10.1055/s-0037-1606316

**Published:** 2017-09-07

**Authors:** Roland Strasser, Thomas Wein, Marc Wieder, Johannes Erhardt, Jörg Kaufmann

**Affiliations:** 1 Department of Orthopaedics, Spital Grabs, St Gallen, Switzerland

**Keywords:** biceps femoris knee joint, rupture, suture

## Abstract

Isolated biceps femoris rupture is a rare injury associated with limitation in the function of the knee. We present a 65-year-old man who sustained an isolated complete rupture of the tendon of the biceps femoris. The diagnostic was reached after clinical examination and magnetic resonance imaging of the affected knee. This case was treated with a surgical tendon reconstruction. The outcome was good and the patient was able to walk normally again without limitation, even if he did not comply with our recommendation.

The biceps femoris has a long and a short head. The proximal long head is inserted at the tuber ischiadicum and the short head at the linea aspera. Distal it inserts at the lateral side of the head of the fibula. It helps in the extension, external rotation of the hip, and flexion of the knee. 1 A rupture of the distal insertion of this muscle is a very rare injury. In the literature, we were only able to find some disparate case reports. 2 3 4 5 Thus, it is in our interest to report this case.

## Case Presentation

A 65-year-old man as he was moving a carpet had stretched his right leg and felt a stabbing pain in the laterodorsal distal tight. He could walk but felt instability in the knee. In the clinical exam, there was a depression in regard to the dorsolateral lateral epicondylus. A strength of two-fifth of the flexion of the knee was also there. There was no intra-articular swelling, no meniscus pathology, no pathological drawer, and the collateral ligaments were stable. An ultrasound was performed but due to the intramuscular hematoma, it was not possible to identify the injury. Therefore, a magnetic resonance imaging (MRI) was performed.

**Fig. 1:**
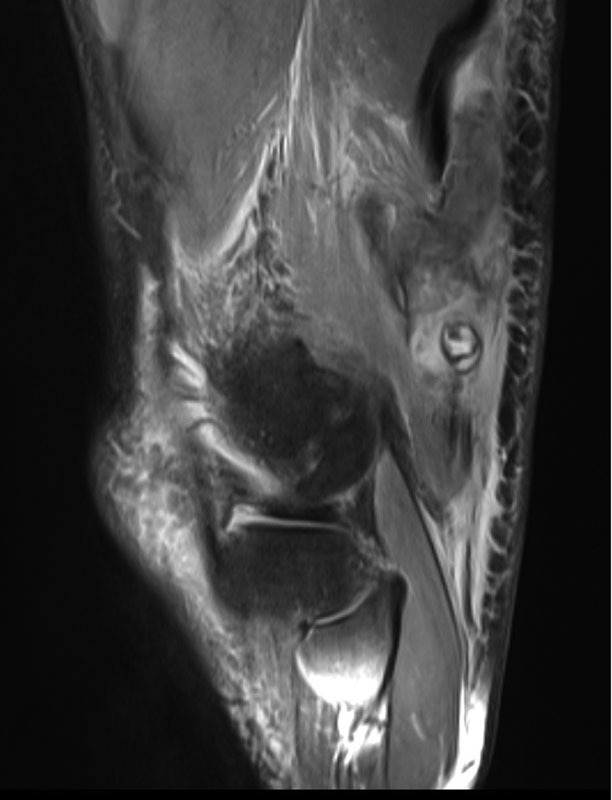
Magnetic resonance imaging finding.

**Fig. 2:**
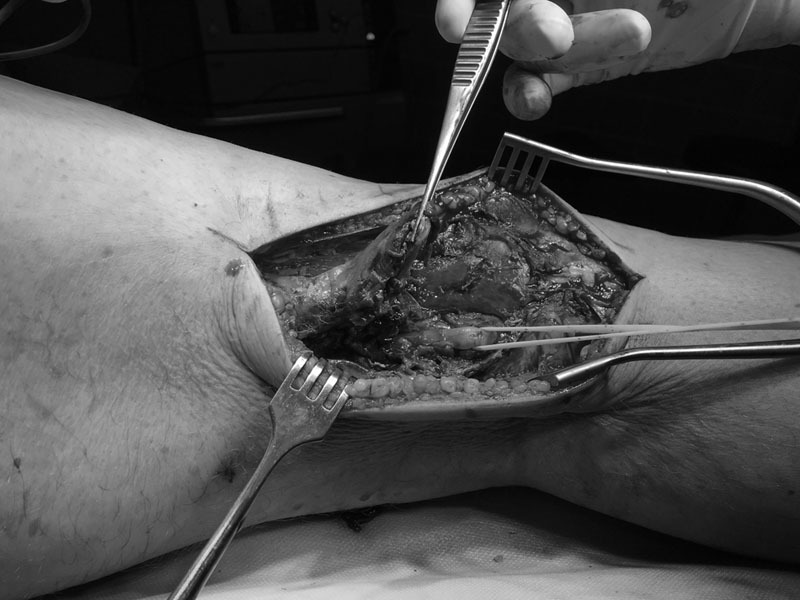
The intraoperative injury.

**Fig. 3:**
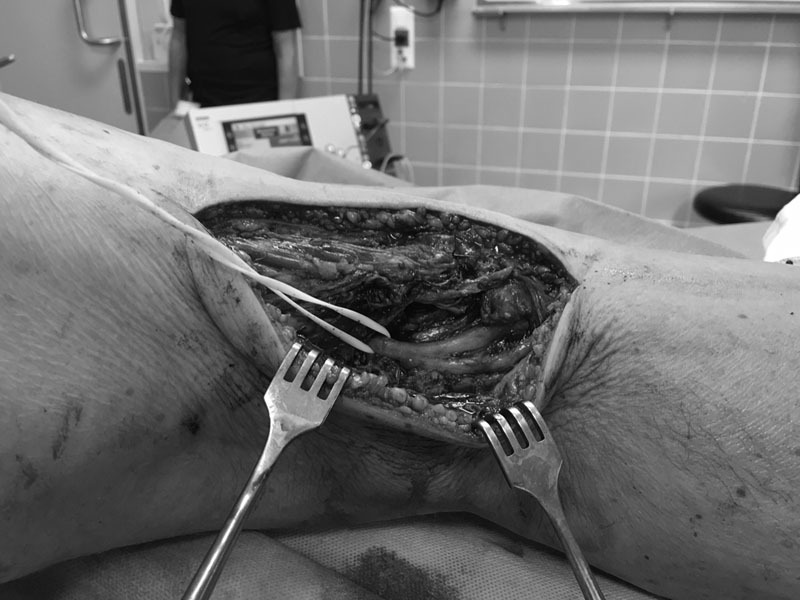
After tendon repair.

On the MRI ( Fig. 1 ), we could follow cranially the distal biceps femoris during 3 cm before a complete rupture of the tendon around the knee. There is a 6-cm dehiscence and tendon retraction with an extended hematoma. The other structures of the knee were intact. With this pathology and the strength deficit, we decided to repair the tendon. After suture with FiberWire of the tendon, we were able to totally stretch the knee with the hip a 0-degree flexion ( Figs. 2 and 3 ). Postoperatively, we limited the knee extension to 30 degrees with a free flexion in a splint for 6 weeks and with only a 20 kg weight bearing.

## Outcome

The patient did not follow the 20 kg weight bearing. After 6 weeks, the patient was able to walk again without limitation or instability. The tendon was difficult to palpate as there was a persistence of a swelling in the operative site.

## Discussion

An isolated rupture of the biceps femoris is extremely rare. It is often associated with complex injury of the knee or injury of the posterolateral capsular ligament complex. 6 Most of the hamstring injury are proximal or in muscle belly. 7 Other isolated distal hamstring injury such as the semimembranosus is even more rare. This can be explained as the biceps femoris is the strongest flexor muscle, biomechanical study measured a 75% loss of strength of the knee flexion by resection of the muscle. 5 With these elements, we could conclude that an operative treatment is the best solution, but as stated previously, there are very few studies about this injury, not enough to have a consensus. There are also studies of a conservative treatment which can lead to full recovery. 5 Kusma et al 8 tried to summarize the outcome of nine case reports, where only one study was treated conservatively. The conservative case had a very good outcome with return to physical activity, and he only had a small flexion deficit. They demonstrate that the injury pattern is from a hyperextension or a flexion against resistance. In some rare case of posterolateral injury is the small head of the biceps femoris tendon used for tenodesis repair. With this surgery, the patient presents a good result, although the flexion function of the muscle is lost. 9

## Conclusion

Till now almost all patients have been treated operatively, and in the literature, we only found two cases of conservative therapy 5 8 ; therefore, it is not possible to compare it to a conservative treatment. We do know that both can work, but as the consequences of nonhealed injury will greatly handicap the patient we would preferably go with the treatment that is more common, therefore the operation. More studies will be required to show us what is the best therapy.
